# Mechanistic study of electroacupuncture in the treatment of insomnia: study protocol for a clinical trial of serum metabolomics based on UPLC-Q/TOF-MS and UPLC-QQQ-MS/MS

**DOI:** 10.3389/fpsyt.2025.1499361

**Published:** 2025-03-25

**Authors:** Yuting Zhang, Ziqiong Cao, Junlan Ye, Guoliang Dai, Shan Qin, Xiaoqiu Wang, Wenzhong Wu, Chengyong Liu

**Affiliations:** Jiangsu Province Hospital of Chinese Medicine, Affiliated Hospital of Nanjing University of Chinese Medicine, Nanjing, China

**Keywords:** insomnia, electroacupuncture, metabolomics, clinical trials, study protocols

## Abstract

**Background:**

Insomnia is the most prevalent sleep disorder worldwide. Electroacupuncture is effective in improving sleep quality, daytime fatigue status, and anxiety and depression in patients with insomnia, and this study aimed to investigate the metabolic pathways and their possible mechanisms in response to the efficacy of electroacupuncture in the treatment of insomnia.

**Methods:**

A single-center, double-blind, clinical trial was the study’s design. For this study, a total of 99 participants were enrolled, and they will be split into two groups: one for insomnia and the other for health. There are 33 healthy people in the healthy group and 66 insomnia patients in the insomnia group. Acupuncture treatment will be administered to the intervention group three times a week for four weeks, for a total of twelve treatments, and will be followed up for 3 months. A combination of UPLC-Q/TOF-MS and UPLC-QQQ-MS/MS was used to qualitatively and quantitatively examine the serum of 99 participants. The Pittsburgh Sleep Quality Index (PSQI) and serum metabolomics provided the primary findings. The Insomnia Severity Index (ISI), Hyperarousal Scale (HAS), Fatigue Feverity Scale (FSS), Hamilton Depression Scale (HAMD), Hamilton Anxiety Scale (HAMA), Sleep Diary and The Montreal Cognitive Assessment (MoCA) were the secondary outcomes. For the insomnia group, serum will be collected at baseline, at the end of treatment, and the scale will be collected at baseline, after 4 weeks of treatment, and at 3 months of follow-up. For the healthy group, serum will be collected at baseline.

**Discussion:**

This study aimed to assess the modulatory effects of electroacupuncture on relevant metabolic markers using serum metabolomics, to explore the potential mechanisms and relevant metabolic pathways of electroacupuncture for the treatment of insomnia, and to provide strong scientific evidence for the treatment of insomnia by electroacupuncture.

**Trial registration:**

ChiCTR2400085660 (China Clinical Trial Registry, http://www.chictr.org.cn, registered on June 14, 2024)

## 1 Introduction

The most common sleep disorder in the world, insomnia is defined by clinically frequent and persistent problems getting to sleep, staying asleep, and/or waking up early. It is also characterized by dysfunction during the day, including weariness or low energy, mood swings, and cognitive decline (1). According to reports, 10% of people fit the diagnostic criteria for insomnia, while an additional 15%–20% of adults sporadically experience the symptoms of insomnia, with the percentage of adults experiencing insomnia rising each (2, 3). Patients’ physical and mental well-being are gravely endangered by insomnia, which also places a significant financial strain on the healthcare system (4). Research has demonstrated that sleeplessness raises the risk of a number of chronic physical illnesses, including diabetes, hypertension, heart disease, cancers, and mental health issues (5–8). The neurobiological mechanisms of insomnia are unknown, and most scholars believe that insomnia occurs as a result of psychological and physiological hyperarousal of the organism. Current diagnostic methods for insomnia rely on subjective patient reports, sleep diaries, and clinical interviews, and lack quick and accurate objective markers, such that insomnia is underdiagnosed in primary care settings thus leading to exacerbation of the condition (9–11). In addition, insomniacs undergoing treatment may lack objective knowledge and suffer from deep cognitive rumination, thus compromising clinical outcomes. Modern medical treatment of insomnia includes medication and cognitive behavioral therapy. However, commonly used sedative and sleeping medications in clinical practice are often accompanied by different degrees of adverse effects, including dizziness, headache, daytime sleepiness, and increased risk of dementia, leading to low patient acceptance (12). Cognitive-behavioral therapy is a first-line therapy recommended by clinical guidelines, which is more difficult to implement clinically due to its lack of professional resources, time-consuming nature, and poor patient compliance (13). Electroacupuncture is effective in treating insomnia with a non-placebo effect (14), but the specific mechanism of its effect is unknown, and its efficacy assessment is mainly based on the patient’s subjective scale, which lacks objective assessment indexes and testing methods.

In order to investigate the pathophysiology of diseases and the mechanisms by which therapies work, metabolic technologies is used to identify the various changes that occur in biological systems under various medical conditions. It also facilitates the rapid and thorough analysis of differential metabolites to elucidate physiological or pathological pathways of metabolism (15). Additionally, the technology explores the relationship between pathophysiological changes and small molecule metabolites. 328 metabolites are thought to be very important in human sleep, and particularly abundant metabolites include carnitine, aromatic amino acids, phosphatidylcholine, and lysophosphatidylcholine (16).Previous studies have explored the unique serum metabolomic profile of insomnia and found that insomnia induces perturbations in metabolites of amino acid metabolism, neurotransmitter metabolism, and glycolipid metabolic pathways (17). One study showed that by collecting plasma from insomnia patients and applying untargeted metabolomics analysis, 64 metabolites were associated with sleep duration, and elevated levels of amino acid metabolites (such as branched-chain amino acids and gamma-glutamyl dipeptide) were associated with later sleep duration (18). In another study, elevated energy metabolites and reduced branched-chain amino acid catabolic metabolites, as well as altered glucose metabolism, were observed in patients with insomnia by comparing insomnia patients with healthy controls, suggesting an increased susceptibility to diabetes in insomnia patients (19). Insomnia leads to significant alterations in specific metabolic pathways, which may be evidence of a link between insomnia and other somatic and psychiatric disorders. Therefore, this study aims to utilize metabolomics techniques to qualitatively and quantitatively analyze the sera of healthy individuals and patients with insomnia, in order to screen for differential metabolites, further explore the possible pathogenesis of insomnia, and search for objective biomarkers for it.

In addition, the development and translational application of metabolomics technology can not only be used to explore the pathogenesis of insomnia but also provide a new platform for constructing therapeutic strategies for insomnia. Studies have found that insomnia patients who take benzodiazepine receptor agonists and benzodiazepines have poor glucose metabolism (20), while exogenous melatonin significantly reduces glucose tolerance and increases insulin resistance in subjects (21). This suggests that medication may have adverse metabolic effects. A preliminary study of electroacupuncture Shenmen-Sanyinjiao acupoint pairing using non-targeted metabolomics techniques found that the Shenmen-Sanyinjiao pairing significantly potentiated serum glycerophosphatidylserine-like metabolites in patients with insomnia, resulting in a significant improvement in the impairment of diurnal functioning in insomnia patients (22). However, the current study lacked quantitative analysis of specific metabolites to observe the dynamic change pattern under electroacupuncture intervention. Therefore, it is necessary to conduct more in-depth studies to provide a scientific basis for the mechanism of action of electroacupuncture.

Ultra-high performance liquid chromatography (UPLC) coupled with mass spectrometry offers the benefits of high resolution, high sensitivity, and high efficiency with the advancement of analytical instruments, enabling quick qualitative and quantitative examination of samples. Furthermore, blood is a frequently utilized specimen in metabolomics technology because it is simple to obtain, has the ability to represent metabolite changes under a variety of intervention and pathophysiological settings, and is a valuable source of disease biomarkers. Based on this, we set up a clinical trial, using ultra high performance liquid chromatogravimetric quadrupole time-of-flight mass spectrometry (UPLC-Q/TOF-MS) and ultra-high performance liquid chromatogravimetric triple quadrupole tandem mass spectrometry (UPLC-QQQ-MS/MS) for qualitative and quantitative analysis of subjects’ serum. To screen out the metabolite differences between healthy people and insomniacs and insomniacs before and after treatment, explore the correlation between pathophysiological changes in insomnia and small molecule metabolites, as well as the metabolic pathway in response to the therapeutic effect of electroacupuncture, and provide scientific evidence for the possible mechanism of electroacupuncture regulating metabolic network in the body to improve insomnia symptoms.

## 2 Materials and methods

### 2.1 Study design

**Figure 1:**
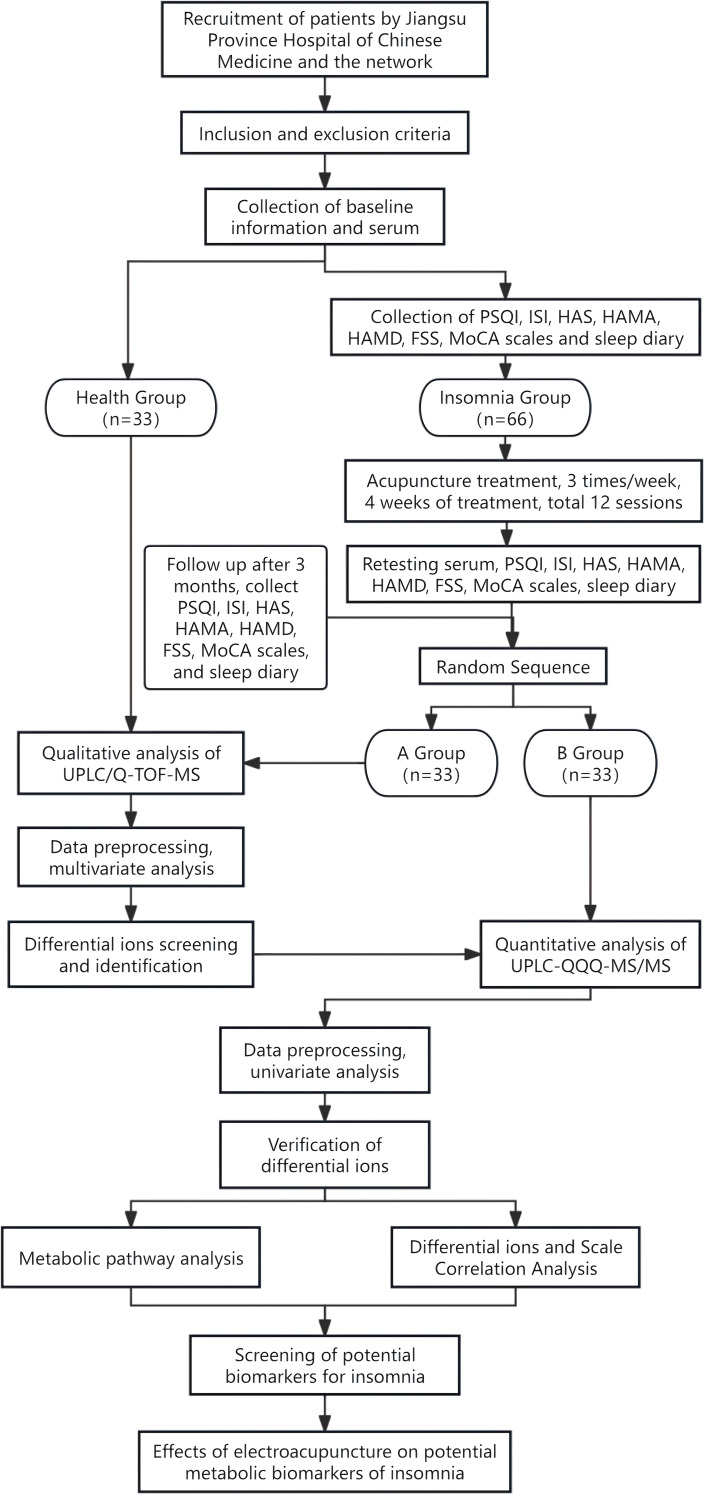
Flow diagram of the trial.

**Figure 2:**
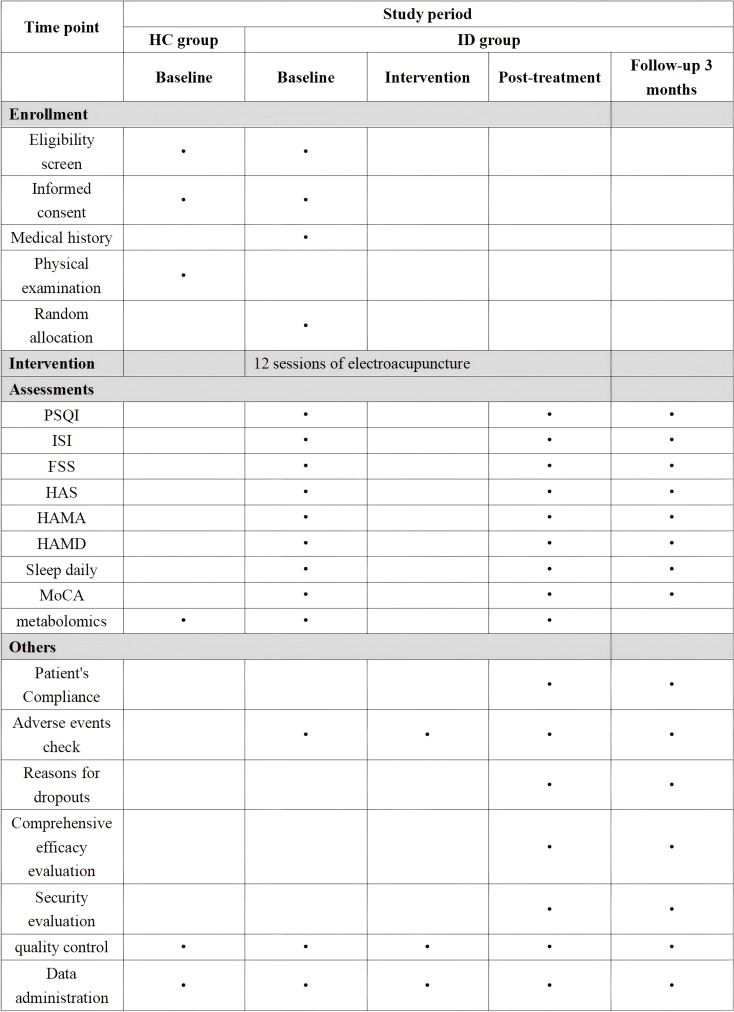
Schedule of enrolment, interventions, and assessments.

The study design is a single-center, double-blind, clinical trial to investigate the metabolic pathways and possible mechanisms underlying the efficacy response of electroacupuncture for insomnia. The study protocol follows the SPIRIT guidelines, 99 subjects will be recruited and divided into an insomnia group and a healthy group. The International Classification of Sleep Disorders, Third Edition (ICSD-3) diagnostic criteria will be used to diagnose subjects with insomnia. Those who meet the inclusion criteria will also sign an informed consent form, complete the following scale assessments, and have their serum collected: PSQI and ISI to measure sleep; HAS to measure hyperarousal state; HAMD and HAMA to measure mental state; and FSS to measure physical and mental fatigue, sleep diary to measure sleep behavior, and MoCA to measure cognition to gather baseline assessment data. This will be followed by 4 weeks of treatment. Retesting of the above scales will be completed at the end of treatment and at 3 months follow-up, as well as serum collection’s will be completed at the end of treatment. The healthy group will complete serum collection at enrollment. The insomnia group will be randomly divided into Group A and Group B, matched with the healthy group. Group A and the healthy group will be screened for differential metabolites using metabolomics analysis with non-targeted UPLC-Q/TOF-MS, and Group B will be quantified for the screened differential metabolites using metabolomics with targeted UPLC-QQQ-MS/MS. The flow chart of the experiment is shown in **Figure 1**. The flow schedule is shown in **Figure 2**.

### 2.2 Participants

#### 2.2.1 Patient recruitment

Patients with insomnia will be recruited in this study in the following three ways: 1) cases will be recruited from the Acupuncture and Rehabilitation Department and Physical Examination Center of Jiangsu Provincial Hospital of Traditional Chinese Medicine (JSTH); 2) recruitment posters will be placed in the outpatient department of Jiangsu Provincial Hospital of Traditional Chinese Medicine (JSTH); 3) WeChat platform will be used to push out the recruitment articles.

#### 2.2.2 Inclusion criteria

##### 2.2.2.1 Inclusion criteria for insomnia patients

1. Age 18-60 years old;
2. Meet ICSD-3 diagnostic criteria;
3. 5 points < PSQI < 17 points;
4. not receiving psychotropic medication;
5. have no communication or cognitive impairment and can perform basic operations on a smartphone;
6. Signed informed consent.

##### 2.2.2.2 Inclusion criteria for healthy people

1. Normal blood count, liver function, kidney function, electrocardiogram, blood glucose, and blood lipid after consultation and health examination; healthy, no previous organic or functional diseases or psychiatric disorders;
2. Be in the age range of 18 to 60;
3. not recently participated in other clinical studies;
4. Voluntarily participate in this trial, agree to and sign the informed consent form.

#### 2.2.3 Exclusion criteria

##### 2.2.3.1 Exclusion criteria for insomnia patients

1. Neuroendocrine, cardiovascular, cerebrovascular, hematopoietic system, tumor and other serious diseases;
2. Sleep disorders caused by depression, anxiety, schizophrenia or other serious mental illnesses with HAMA score >14 and/or HAMD score >17;
3. Other sleep disorder disorders such as obstructive sleep apnea, rapid eye movement (REM) sleep disorder or restless legs syndrome;
4. Those who are not suitable to receive acupuncture treatment, such as those who are pregnant or breastfeeding;
5. Those who have received acupuncture treatment for insomnia in the past month;
6. those with alcohol and/or other drug abuse or dependence.

##### 2.2.3.2 Exclusion criteria for healthy people

1. Pregnant and lactating women;
2. Alcoholics and smokers.

#### 2.2.4 Shedding criteria

1. Patients refused to cooperate with the trial after enrollment;
2. Patients withdrew on their own during the study or did not complete the entire course of treatment and had poor compliance;
3. Losing contact and moving to other places during treatment, and unable to cooperate with the subsequent clinical observation.

#### 2.2.5 Discontinuation criteria

1. Inability to tolerate needling or allergic reactions;
2. Serious adverse events during treatment, or serious complications caused by other diseases;
3. Female patients found to be pregnant.

### 2.3 Sample size

PASS 15.0 software was used to determine the sample size based on the nature of the study, with the control group being the healthy group and the treatment group being the insomnia group. Patients with insomnia use the PSQI score as an observed outcome indicator. Based on the pre-test results and literature review, the mean PSQI score for the drug control group is 10.76 ± 3.32 points, and it is anticipated that this score will decrease by 2.63 points for the insomnia group. The effect size (Effect Size) of the two groups is 0.8, α=0.05, Power=0.9, and this means that the sample size for the treatment group is 52 cases, and the sample size for the health group is 26 cases; taking into account variables like fewer visits, an increase in cases, and an increase in cases. The sample size for the treatment group was 52 cases, and the sample size for the healthy group was 26 instances. After taking lost visitation into account, the sample size was expanded by 15%, resulting in 66 cases for the treatment group and 33 cases for the healthy group in this experiment.

### 2.4 Ethics and trial registration

The ethical and informed consent materials for this study protocol were approved by the Ethics Committee of the Affiliated Hospital of Nanjing University of Traditional Chinese Medicine (Jiangsu Provincial Hospital of Traditional Chinese Medicine) on June 13, 2024, with the Committee Approval No. 2024NL-124-02. The study was likewise registered in the China Clinical Trial Registry, with Approval No. ChiCTR2400085660.The study was conducted with the approval of the China Clinical Trial Registry (CCTR).

### 2.5 Randomization

Following screening according to inclusion and exclusion criteria, 33 eligible patients with insomnia will be split into two groups, A and B, using whole cluster randomization, with a 1:1 ratio. Researchers not participating in the experimental implementation will create the randomized sequences and use IBM’s SPSS 25.0 software (IBM, Armonk, NY, USA) for statistical analysis. The randomized sequences will be inserted into opaque envelopes in the order of inclusion using the envelope method of concealment. Patients who fulfilled the requirements were given envelopes in the order of enrollment to be given to the data analyst, who then obtained the patient groups. An individual else was in charge of patient inclusion.

### 2.6 Blinding

By the principle of blinding, the grouping results will be blinded to the participants, acupuncturists, and evaluators. Because the acupuncture protocol was consistent in the insomnia group of this study, the acupuncturists do not need to know the grouping results and the acupuncturists cannot assess the results of the intervention. This study will be blinded to the evaluators, who will not be informed of the subgroups, will only be responsible for collecting data from the subjects, and will not be allowed to discuss the treatment with the evaluators. Because the insomnia group of this graduate student will be analyzed using a different metabolomics technique, this study will not be blinded to the metabolomics technique, and data analysts, who will be asked to perform the analysis according to their assigned group will be responsible for statistical analysis and data interpretation.

### 2.7 Intervention

Electroacupuncture will be performed by the same licensed acupuncturist with 5 years of clinical experience in acupuncture. Prior to the trial, the acupuncturist will receive specific training on the treatment process (including the angle and depth of the needles, needle retention time, and needle insertion, travelling, and exit techniques) and will follow a standardized acupuncture protocol. A supervisory team will also be set up to monitor the acupuncturist’s acupuncture practice on site, and if any non-compliance with the protocol is detected, the acupuncturist will be corrected in a timely manner and the situation will be recorded.

#### 2.7.1 Insomnia group

##### 2.7.1.1 Sleep hygiene preaching

Patients will be instructed to pay attention to pertinent precautions at the time of enrollment, including good sleeping environments, such as indoor temperature, light, and humidity; regular work and rest; avoiding the consumption of excitatory food that affects sleep; and refraining from engaging in strenuous exercise before going to bed. The insomnia group received a unified sleep hygiene promotion.

##### 2.7.1.2 Electro-acupuncture intervention

**Figure 3:**
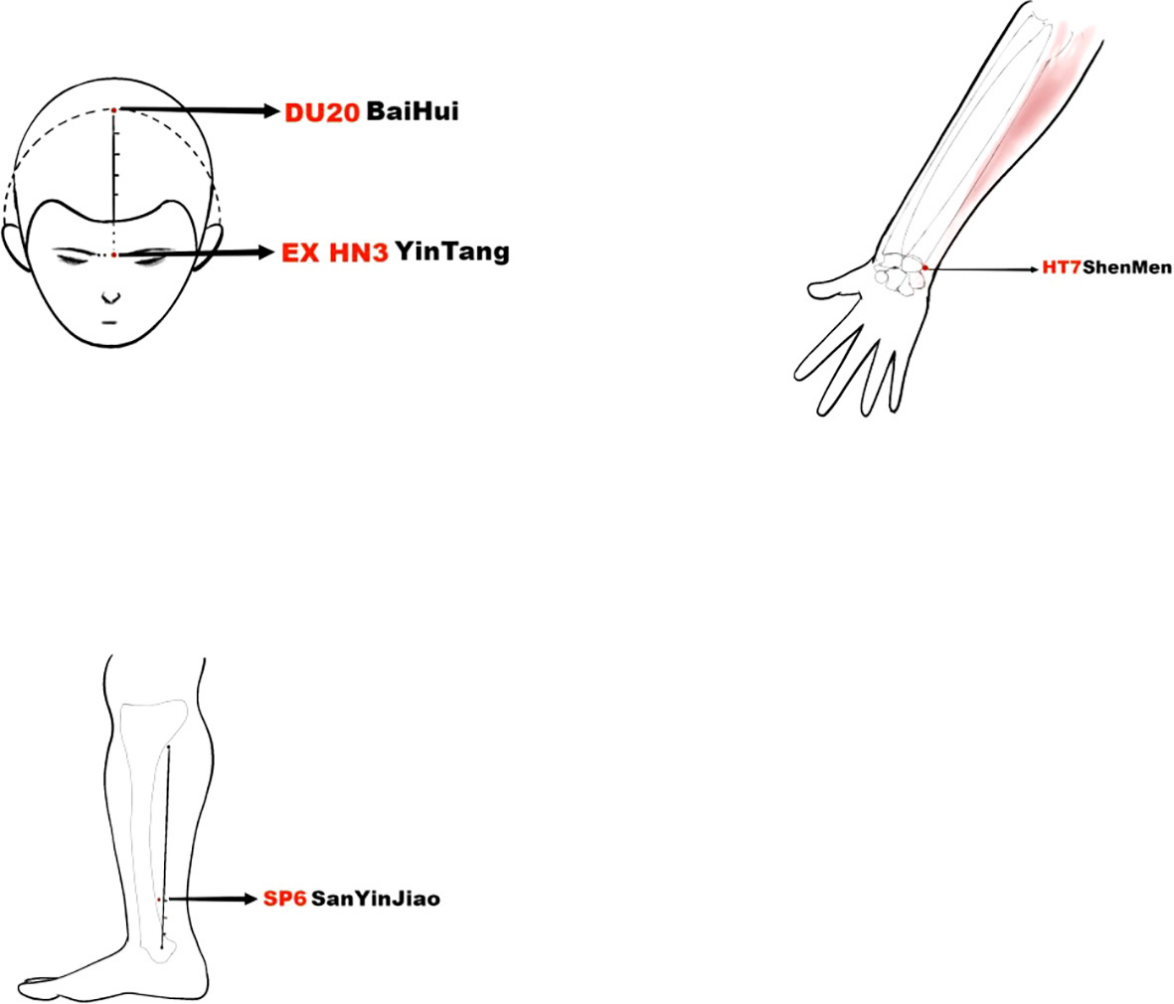
The accurate positioning of acupuncture points.

Insomnia patients will be treated with electro-acupuncture every other day, three times a week for four weeks, for a total of 12 times. Treatment will be carried out at Baihui, Yintang, Shenmen (double), and Sanyinjiao (double), and the positioning will be in strict accordance with the National Standard of the People’s Republic of China: Meridian Point Sites (GB12346-90) (**Figure 3**). Subjects will be placed in the prone position, and disposable sterile acupuncture needles (0.30 × 40 mm, Suzhou Medical Supplies Factory Co., Ltd.) will be used for needle insertion after routine sterilization. For the Baihui acupoint, the needle will be inserted at a 15° angle to the skin, to a depth of 0.5 - 0.8 inches. For the Yintang acupoint, the needle will be inserted at approximately a 15° or smaller angle, using a transverse technique, to a depth of 0.3 - 0.5 inches. For the Shenmen acupoint, the needle will be inserted perpendicularly (90°) to the skin, to a depth of 0.3 - 0.5 inches. For the Sanyinjiao acupoint, the needle will be inserted perpendicularly (90°) to the skin, to a depth of 1 - 1.5 inches. The acupuncture needle was inserted into the acupuncture point and lifted and twisted to achieve the state of getting qi. The low-frequency pulse electroacupuncture therapeutic instrument (Model XS-998B04 Low-Frequency Pulse Electroacupuncture Therapeutic Instrument, Nanjing Komatsu Medical Instrument Research Institute, Nanjing, China) will be connected to the needle handles of the Baihui and Yindang acupoints with a continuous wave pattern, a fixed frequency of 2Hz, and the stimulation intensity will be as tolerated by the subjects, and the needles will be activated after 30min of continuous stimulation.

#### 2.7.2 Healthy group

The healthy group will not receive any treatment.

### 2.8 Outcome assessment

#### 2.8.1 Primary outcome

##### 2.8.1.1 PSQI

A questionnaire was used to evaluate the quality of sleep in a population, including 7 components: sleep quality, time to sleep, sleep duration, sleep efficiency, sleep disorders, hypnotic medications, and daytime dysfunction. The total score of the PSQI is 0-21, and the level of the score is inversely related to the quality of sleep, i.e., the higher PSQI score suggests a worse quality of sleep. A score of ≥5 on the PSQI indicates that clinical treatment may be needed, and the present study used a PSQI score of 5 < PSQI < 17 points as the inclusion criteria for insomnia.

##### 2.8.1.2 Metabolomics

Serum will be collected from the insomnia and healthy groups. Biomarkers associated with electroacupuncture treatment in insomnia patients will be identified through analysis and testing. In addition, the KEGG database will be used to analyze the metabolic pathways associated with these biomarkers, which will be used to explore the metabolic pathways involved in electroacupuncture for insomnia.

#### 2.8.2 Secondary outcome

##### 2.8.2.1 ISI

The purpose of the ISI is to assess an individual’s subjective perception of insomnia on a seven-question, five-point scale that participants fill out based on their sleep over the last week, with higher scores indicating more severe insomnia and greater distress to the individual. Scores range from 0-28. A total score of 0-7 indicates no insomnia distress, 8-14 indicates insomnia distress within the critical range, 15-21 indicates moderate insomnia distress, and 22-28 indicates severe insomnia distress.

##### 2.8.2.2 HAS

HAS is used to evaluate the degree of trait arousal in the waking state of the patient, there are 26 items, and each item is scored 0-3 points, the total score is 0-78 points, the higher the score the higher level of cortical arousal of the patient. HAS > 32 points is considered to be the existence of an excessive arousal state.

##### 2.8.2.3 FSS

The FSS is used to assess the level of fatigue. It consists of 9 entries evaluated at 7 score points, with a gradual transition from a score of 1 to 7 as strongly disagree to strongly agree. A total score below 36 indicates that fatigue may not be present, and a total score above 36 indicates that further evaluation may be required.

##### 2.8.2.4 HAMA

HAMA is used to assess the severity of the patient’s anxiety symptoms and consists of 14 entries with a score of 0-4 for each item and a total score of 0-56, with higher scores representing more severe anxiety in the patient. HAMA score >14 is anxious and excluded.

##### 2.8.2.5 HAMD

HAMD is used to assess the severity of the patient’s depressive symptoms and consists of 17 entries with a score of 0-4 for each item and a total score of 0-68, with higher scores representing a more severe level of depression. HAMA >17 is depressive and is excluded.

##### 2.8.2.6 Sleep diary

Sleep diary, a standard subjective assessment tool for insomnia, is used to observe the severity of insomnia in patients and monitor sleep improvement during treatment, as well as a form of behavioral therapy. It records sleep information such as the previous night’s time of falling asleep, sleep latency, time of waking up in the morning, time of waking up, number and duration of waking up after falling asleep, total sleep time, time of falling asleep, and use of sedative-hypnotic drugs.

##### 2.8.2.7 MoCA

The Montreal Cognitive Assessment (MoCA) is a 30-point scale that takes about 10 min to complete and assesses various dimensions of cognitive functioning: short-term memory, visuospatial executive ability, executive functioning, attention and working memory, language, and orientation in several dimensions of cognitive assessment. Years of education ≤12 scored an additional 1 point to correct for the effect of literacy. The higher the score, the better the cognitive function. A total score of ≥26 is considered normal.

#### 2.8.3 Assessment time point

The insomnia group will have the above information completed at enrollment and after 12 treatments, the scale will be collected again at 3 months follow up, and the health group will have serum collected only at the first enrollment.

### 2.9 Metabolomics

#### 2.9.1 Sample collection

##### 2.9.1.1 Requirements for blood sample collection

1. No caffeinated beverages or food (e.g., tea, chocolate, coffee, cola, etc.), no alcohol, and no food containing cheese and preservatives for 24h until sampling is completed;
2. Avoid strenuous physical exercise for 24 hours before blood collection until sampling is completed;
3. Avoid emotional stress on the day of blood collection and rest for at least 5 minutes before blood collection;
4. Female subjects should avoid menstruation.

##### 2.9.1.2 Collection and storage of blood samples

Venous blood was collected from the subjects, whole blood was collected in serum tubes, and the volume of blood collected was about 4mL, centrifuged at 4°C, 3500r/min for 10min. The upper layer of clear serum was extracted with a pipette gun to 400μL into 1.5ml graduated EP tubes with pointed bottoms, and then placed in a refrigerator at -80°C for storage and preparation for examination.

#### 2.9.2 Metabolomics analysis

##### 2.9.2.1 Metabolite extraction

Metabolite extraction was performed by the organic reagent protein precipitation method. Extraction method: take the cryopreserved serum samples, sorted according to the order of uptake, static thawing until completely liquid; take 40 μL of each sample and add it to the corresponding 96-well plate, add 120 μL of pre-cooled methanol, seal the membrane, shake and mix for 1 min, and place it in the refrigerator at -20 °C overnight; take it out on the next day, reconstitute the dissolution, and centrifuge it for 30 min under the condition of 4 °C, 4000 rpm, and then take 25 μL of the supernatant into a new 96-well plate, add 225 μL of 50% methanol for dilution, and then take it out on the following day. Take 25 μL of supernatant in a new 96-well plate, add 225 μL of 50% methanol for dilution, each take 60 μL of supernatant transferred to a new 96-well microtiter plate, according to the order of the uploading sequence, sealing the membrane marking, and ready for inspection. Take 20μL from each prepared sample and mix it into a prepared quality control sample (hereinafter referred to as “QC sample”).

#### 2.9.3 Chromatography and mass spectrometry conditions

##### 2.9.3.1 UPLC-Q-TOF-MS analysis

###### 2.9.3.1.1 Chromatographic conditions

ACE Excel3 C18column (2.1 mm x 100 mm, 1.8 μm) was used; mobile phase: 0.1% formic acid in water (A)-0.1% formic acid in acetonitrile (B), gradient elution (0~2.5 min, 85%→80%A; 2.5~7 min, 80%-75%A; 7~12 min, 75%→50%A; 12~19 min, 50%→50%A; 12~19 min, 50%→50%A; 12~19 min, 50%→20%A; 19~23 min, 20%→54%A; 23~26 min, 54%→85%A; 26~35 min, 85%→85%A); Flow rate: 0.3 mL-min-1; Column temperature: 40 °C; Inlet volume: 5 μL; Inlet chamber temperature: 4°C.

###### 2.9.3.1.2 Mass spectrometry conditions

the electrospray ionization (ESI) source was used to collect data in positive and negative ion modes; the parameters of the TOF-MS scanning mode were set as follows: relative molecular mass (RMM) scanning range of m/z50~1,000; accumulation time of 0.1500 s; ionization temperature (TEM) of 550 °C; nebulization gas (GS1) of 55 psi; auxiliary heated gas (GS2) of 55 psi; curtain gas (CUR) of 30 psi; de-clustering voltage (DP) 80 V; collision energy (CE) 5 eV; spray voltage (ISVF) in positive and negative ion modes were 5500, 4500 V. Data-dependent scanning (IDA), intelligent dynamic background subtraction (DBS), and high-sensitivity mass spectrometry data acquisition modes were used. The relative molecular mass (RMM) scanning range of the ion scanning mode was m/z50~1,000; the collision energy was (30 ± 15) ev, and the other main parameters were the same as those of the TOF-MS mode.

##### 2.9.3.2 UPLC-QQQ-MS/MS analysis

###### 2.9.3.2.1 Chromatographic conditions

ACE 5 C18-AR (4.6 mmx250 mm, 5 um); mobile phase: acetonitrile (A)-0.1% aqueous phosphoric acid (B), gradient elution (0~10 min, 2%→5%A; 10~20 min, 5%→20%A; 20~35 min, 20%→28%A; 35~40 min, 28%→30%A; 40 ~48 min, 30%→38%A; 48~50 min, 38%→2%A); detection wavelength: 280 nm; Flow rate: 1.0 mL-min-1; Column temperature: 40 °C; Injection volume: 20 μL.

###### 2.9.3.2.2 Mass spectrometry conditions

electrospray ionization (ESI) source, negative ionization (ESI) mode; ion source temperature 350°C; spray voltage -3500 V; multiple reaction monitoring (MRM) mode: in collision gas mode; air curtain gas pressure 0.17 MPa; spray gas, pressure 0.34 MPa; auxiliary heating gas pressure 0.38 MPa.

###### 2.9.3.2.3 Data preprocessing

The UPLC/Q-TOF-MS system operating software MassLynx (SCN633) was used to preprocess the raw data related to metabolomics. The program automatically carried out pre-processing tasks like noise filtering, mass spectral peak identification, and normalization. Ultimately, a three-dimensional data matrix that included retention time, mass-to-charge ratio, and spectral peak area was produced.

###### 2.9.3.2.4 Multivariate statistical analysis

Using MetaboAnalyst 4.0 software (http://www.metaboanalyst.ca), multivariate statistical analyses, such as principal component analysis (PCA), partial least squares discriminant analysis (PLS-DA), and orthogonal partial least squares discriminant analysis (OPLS-DA), were carried out on the obtained three-dimensional data matrices to perform differential metabolite analysis. PLS-DA and OPLS-DA are supervised data dimensionality reduction models. PCR is an unsupervised model that can compress the data into a few main variables to characterize the data. It is primarily used to observe the distribution of each group of samples in their natural state and the overall differences. The variable weight value (variable important in the projection, VIP) in the OPLS-DA model can be used to determine which metabolites are different between the two groups, and metabolites with VIP>1 are typically considered differential metabolites. Multidimensional statistical methods due to the simultaneous influence of correlation and intra-group and inter-group variance and other factors, sometimes the differentially expressed variables found may not be very significant differences in a single dimension, so it is also necessary to validate at the unidimensional statistical level (two-sided/test, p < 0.05) as a way to further screen metabolic markers. The relative contents of the various samples can be hierarchically clustered based on the differential metabolites tested in the samples; the outcomes are displayed as a heat map. Lastly, by comparing the serum relative content levels of the screened metabolic indicators with the study’s PSQI scale scores, including follow-up data, Spearman’s rank correlation analysis was carried out for each metabolic marker.

###### 2.9.3.2.5 Metabolic pathway analysis

The primary mass spectra (MS1) and secondary mass spectra (MS2) information of the metabolites were matched with the metabolites in the HMDB database (http://www.hmdb.ca), KEGG database (http://www.genome.jp/kegg) and METLIN database for identification. The mass error was set to 10 ppm for MS1 and 15 ppm for MS2. MetaboAnalyst 4.0 software was used for the enrichment analysis of metabolic pathways in insomnia based on the KEGG database.

### 2.10 Adverse events and safety assessment

All subjects will be evaluated for safety before and at the end of treatment, and adverse events(AEs) during treatment will be recorded in a timely manner, including local haematomas, needle fainting and stagnation. Subjects will undergo a thorough history review and physical examination prior to treatment to rule out contraindications to the use of electroacupuncture, such as bleeding disorders, severe heart disease, etc. The treating acupuncturist will be rigorously trained in proper electroacupuncture techniques and emergency management, and have extensive clinical experience. In addition, the acupuncturist will check the electroacupuncture before using it to ensure that the electroacupuncture instrument used is working properly, that there are no abnormal alarm messages, and that the electrodes and wires are not damaged. Once subjects are found to have serious discomfort or suspected serious AEs, electroacupuncture treatment will be stopped immediately. First aid equipment and medication are available at the study site, and emergency contact will be established with the hospital emergency department in the first instance so that subjects can be quickly transferred for professional treatment. Serious AEs will be reported immediately to the principal investigator, and we will determine their causes and analyze their relevance to acupuncture in order to assess their impact on subject safety and the need to adjust the study protocol. Long-term follow-up will be arranged for subjects who experience serious AEs to monitor the recovery of their health status.

Safety evaluation was performed according to the grading of adverse reactions: Grade 1: safe, without any adverse reactions; Grade 2: relatively safe, if there are adverse reactions, the treatment can be continued without any treatment; Grade 3: there are safety problems, with moderate adverse reactions, the treatment can be continued after doing treatment; Grade 4: the trial was discontinued due to adverse reactions.

### 2.11 Quality control

PSQI, ISI, HAS, FSS, HAMA, HAMD, and MoCA scales will be evaluated by professionally qualified physicians. Standard operating procedures and quality control procedures for serum metabolomics using UPLC-Q/TOF-MS and UPLC-QQQ-MS/MS will be established, and the data will be analyzed and processed by professional instruments to ensure the accuracy of the data. The investigators signed an investigator’s declaration, received training on the trial procedure, and had their quantitative criteria for symptoms and indications validated for consistency prior to the project’s launch. In order for the subjects to cooperate with the experiment and completely grasp its requirements, the investigator must properly carry out the informed consent process. The needle counting method was used to monitor the subjects’ needle compliance. Compliance = (actual number of needles/supposed number of needles) × 100%. Compliance <80% or >120% was considered a major violation of the trial protocol.

### 2.12 Data management

Data will be recorded in paper form on the CRF by the assessor. All raw data will be kept in the Affiliated Hospital of Nanjing University of Traditional Chinese Medicine under the supervision of the Ethics Committee of the Affiliated Hospital of Nanjing University of Traditional Chinese Medicine. In order to guarantee that the data records are accurate, timely, comprehensive, and truthful, the investigators must write the study medical records concurrently with treating the individuals. For data reporting using e-CRF, a person will be designated as the “e-CRF entry officer”, who will be responsible for the initial review of the study charts and logging in to fill out the e-CRF. Upon receipt of the study charts, the “eCRF entry clerk” must first review the study charts for completeness of the project records, and then file two copies of the eCRF. The researcher will collect the data and create a database that will summarize and analyze the data recorded in the study charts to generate the conclusions and the report of the study. Upon completion of the study, the database will be locked for security purposes. Participants’ personal information will be considered confidential. Documents related to quality control, such as original records of data consistency checks, numerical range and logic checks, and blinded audits, will be retained.

### 2.13 Statistical analysis

In this study, the software SPSS25.0 was first used to process the experimental data, and the measurement data were expressed by `x ± s, and the count data were expressed by the rate or the constitutive ratio. Comparison of the measurement data between the two groups was firstly analyzed by normality analysis, a t-test was used for the measurement data that conformed to normality and chi-square, and a non-parametric rank-sum test was used for the measurement data that did not conform to the normal distribution; chi-square or Fisher’s exact probability method was used for the count data. p<0.01 was taken as the difference was significant; p<0.05 was taken as the difference was statistically significant between the two groups.

### 2.14 Trail Status

Protocol: version 2.0, 5/6/2024. Subjects are currently being recruited for this study.

## 3 Discussion

The present study is a single-center, double-blind, clinical trial aimed at assessing the efficacy of electroacupuncture using a combined metabolomics scale and exploring the possible efficacy mechanisms and related metabolic pathways of electroacupuncture in the treatment of insomnia.

Metabolic processes and sleep are intimately associated, and both are present in all living things. Sleep disorders and deprivation can have an impact on metabolism since sleep controls bodily activities and is necessary to maintain a normal metabolism. According to recent study, insomnia can cause metabolic dysregulation by impairing insulin sensitivity, glucose tolerance, and energy balance, as well as by causing inflammation (23). According to research on animals, persistent stress-induced sleeplessness in mice results in the activation of orexin neurons in the lateral hypothalamus, which leads to hyperphagia and a decrease in the leptin/growth factor-releasing peptide ratio (24). On the other hand, mice fed a high-fat diet and subjected to prolonged stress have higher levels of free fatty acids and glucose in their blood, as well as more metabolic dysfunction including aberrant glucose tolerance (25). In addition, it was found that insomniac Drosophila showed elevated dopamine concentrations as well as elevated levels of triglycerides, cholesterol, and free fatty acids (26). The aforementioned research all point to a tight connection between metabolic abnormalities and the etiology of insomnia. In line with the hyperarousal theory of insomnia, clinical research has also revealed a 6-9% rise in 24-hour metabolic rate in patients with insomnia compared to healthy people (27).Additionally, an enlarged hypothalamic-pituitary-adrenal (HPA) axis can lead to food cravings and overeating, which can worsen the condition of the axis and raise the risk of developing other metabolic disorders such obesity, diabetes, and hypertension (28). Therefore, discovering the physiological and molecular processes involved in sleep regulation and metabolic balance can play an essential role in enhancing sleep and metabolic function in individuals.

Acupuncture, as a representative of traditional non-pharmacological therapies in Chinese medicine, is clinically appropriate for insomnia (29). Previous clinical studies have confirmed that electroacupuncture can effectively improve sleep quality, daytime fatigue status, and anxiety and depression in patients with insomnia (30), but the study of the mechanism of electroacupuncture in the treatment of insomnia has been the focus of most scholars. Some studies have found that acupuncture may improve the sleep quality of insomnia patients by increasing the serum level of 5 hydroxytryptamine and decreasing the level of norepinephrine and dopamine in insomnia patients (31). The team’s previous clinical studies have also shown that electroacupuncture can improve sleep quality and hyperarousal in insomnia patients by regulating their serum HPA axis and GABA levels (32, 33). In addition, some studies have shown that the concentrations of IL-1β, IL-6, and TNF-α in the hypothalamus of rats with insomnia model were reduced, and electroacupuncture treatment could significantly increase the concentrations of IL-1β, IL-6, and TNF-α in the hypothalamus of rats with insomnia model and improve the inflammatory response (34). This shows that the effect of electroacupuncture is not simply the regulation of a transmitter or a substance, but an effect produced by the synergistic action of multiple regulations. Therefore, the use of a holistic and comprehensive observation method is of great significance for the systematic interpretation of the material basis and mechanism of electroacupuncture efficacy. Metabolomics fits the holistic view of Chinese medicine theory and the thinking method of “to look outside and look inside, to see the small and know the big picture”, as well as the overall regulation of electroacupuncture, which can help to reveal the mechanism of electroacupuncture’s action. In this study, we will firstly use UPLC-Q/TOF-MS technology for untargeted metabolomics analysis to screen out the differential metabolites associated with insomnia from the serum of insomnia patients by comparing the insomnia group with healthy individuals, and further analyze the MetaboAnalyst 4.0 database to identify the important metabolic pathways and major metabolic routes. Next, by comparing the insomnia patients before and after acupuncture treatment, we will observe whether these abnormal changes are significantly dialed back or not, as a way to prove that acupuncture can improve insomnia symptoms by regulating these metabolic pathways. In addition, this study will apply the UPLC-QQQ-MS/MS technique to accurately quantify the key differential metabolites to further validate the UPLC-Q/TOF-MS screening results. By mutual validation of the two techniques, we will ensure the accuracy and reliability of the screened differential metabolites.

There are some limitations of this study. First, the follow-up period of this study was short due to limited conditions. However, because most insomnia patients have a long duration of illness and are difficult to cure, it is necessary to observe the long-term effects of acupuncture on regulating metabolism and improving sleep quality. Secondly, the research employed a single-center design, which may introduce experimental bias and result in a single sample that lacks representativeness. More influencing factors should be considered and the results should be further validated and explored in a large sample population. Finally, the serum collected from subjects in this study was not fasting serum and may be affected by more factors, which may lead to bias and thus affect the results. Therefore, further metabolic studies with multicenter, large samples and extended follow-up periods are needed in the future.
